# Case Report: The many faces of bullous pemphigoid

**DOI:** 10.3389/fimmu.2023.1272742

**Published:** 2023-10-12

**Authors:** Laura Rechtien, Lukas Sollfrank, Yannick Foerster, Carola Berking, Michael Sticherling

**Affiliations:** ^1^ Department of Dermatology, Uniklinikum Erlangen, Friedrich-Alexander-Universität Erlangen-Nürnberg, Erlangen, Germany; ^2^ Deutsches Zentrum Immuntherapie (DZI), Friedrich-Alexander-Universität Erlangen-Nürnberg, Erlangen, Germany

**Keywords:** bullous pemphigoid, juvenile pemphigoid, pemphigoid gestationis, bullous autoimmune disease, COVID-19 vaccination

## Abstract

The pemphigoid group comprises a number of bullous skin diseases with autoantibodies against different constituents of the basement membrane zone that result in subepidermal detachment and clinically characteristic tense blisters, erosions, urticarial erythema, and itching. Apart from the most frequent type of bullous pemphigoid with antibodies against BP180, which is found predominantly in elderly patients, the disease may present at other ages and different pathogenic conditions. Here, four cases are presented of young age (3 months and 25, 34, and 46 years) and in association with vaccination, pregnancy, or metastatic cancer. Though anti-BP180 was found in all cases, a different pathogenic background may be found in any of them, resulting in characteristic clinical manifestation, yet demanding specifically adapted therapeutic approaches.

## Introduction

Bullous pemphigoid (BP) is the most common human autoimmune bullous skin disease with an incidence of 7/100,000 inhabitants/year. It occurs predominantly at the beginning of the 7th decade of life. Autoantibodies are found against various constituents of the basement membrane zone, which induce subepidermal detachment and an inflammatory cell infiltrate in the superficial dermis containing eosinophils (1). Clinically, BP is characterized by tense blisters on normal or erythematous surrounding skin, erosions, urticarial lesions, and itching and characterizes a set of diseases currently referred to as the pemphigoid group (2). Among other antigens like p200 and α4β6 laminin, bullous pemphigoid antigen 180 kD (BP180), representing collagen XVII, is the main immunogenic target. It characterizes the most frequent subset of pemphigoid diseases, BP in the strict sense (3). The different antibody subsets seem to characterize clinical manifestations, which vary regarding prognosis, predilection sites, associated malignancy, and therapeutic responses. Specifically, linear IgA dermatosis and pemphigoid gestationis are related to different immunoglobulin responses (IgA) or target structures different from the most immunogenic NC16A part of BP180, which characterizes classical BP. In this case series, rare subsets of pemphigoid disease are described and discussed, relating them to their role within the group of pemphigoid diseases.

## Case histories

**Figure 1:**
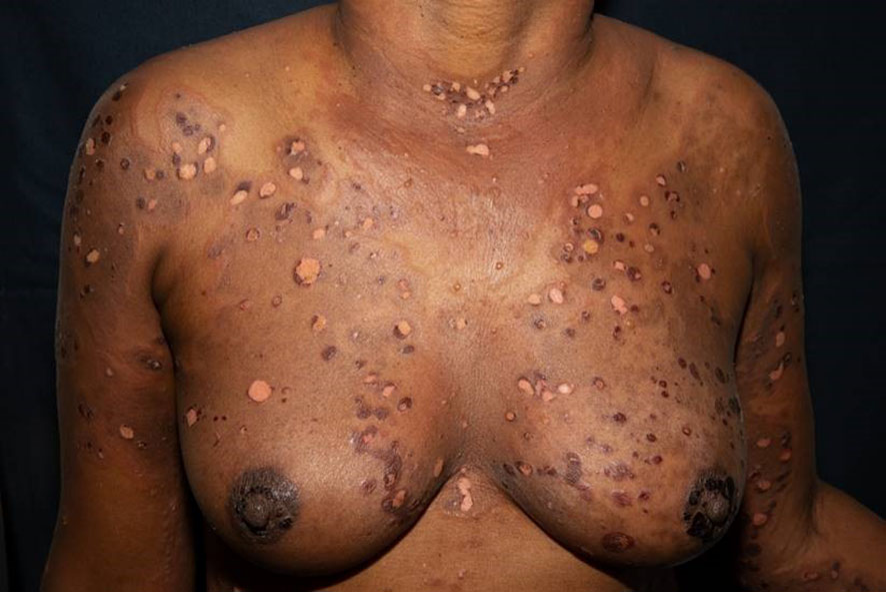
Multiple erythematous, partly excoriated papules, blisters, and erosions disseminated on the entire skin.

The first case shows a 43-year-old female patient, Fitzpatrick skin type VI, who developed marked pruritus 5 days after her first COVID-19 vaccination (mRNA, Pfizer BioNTech). Multiple erythematous, partly excoriated papules as well as blisters and erosions were disseminated on the entire skin (**Figure 1**). Despite local therapy with mometasone furoate cream, the lesions progressed. The patient had no previous medical history, no allergies, no comedication or consumption of any other noxious substances. BP was confirmed by histologic and direct immunofluorescence as well as immunoserologic examinations. The tumor search including abdominal ultrasound, chest X-ray, and hemoccult remained without pathologic findings. Dapsone p.o. and prednisolone 1 mg/kg body weight was initiated, but had to be discontinued due to methemoglobinemia, and azathioprine p.o. was stopped due to toxic hepatitis. The patient ultimately received intravenous immunoglobulins (IVIGs) (Intratect^®^), mycophenolate mofetil p.o., and prednisolone p.o. During further course, the patient developed steroid-induced diabetes mellitus type 2, which was treated with metformin. Incipient arterial hypertension was controlled with ramipril. The patient is currently on mycophenolate mofetil 2 g/day and prednisolone 5 mg/day p.o. as well as clobetasol proprionate cream topically without any relapses. Because of the pronounced skin findings, we directly opted for a combined antibiotic and anti-inflammatory systemic treatment with dapsone and prednisolone to accompany the local therapy. The patient has been followed up since diagnosis, currently for 1 year and 10 months.

**Figure 2:**
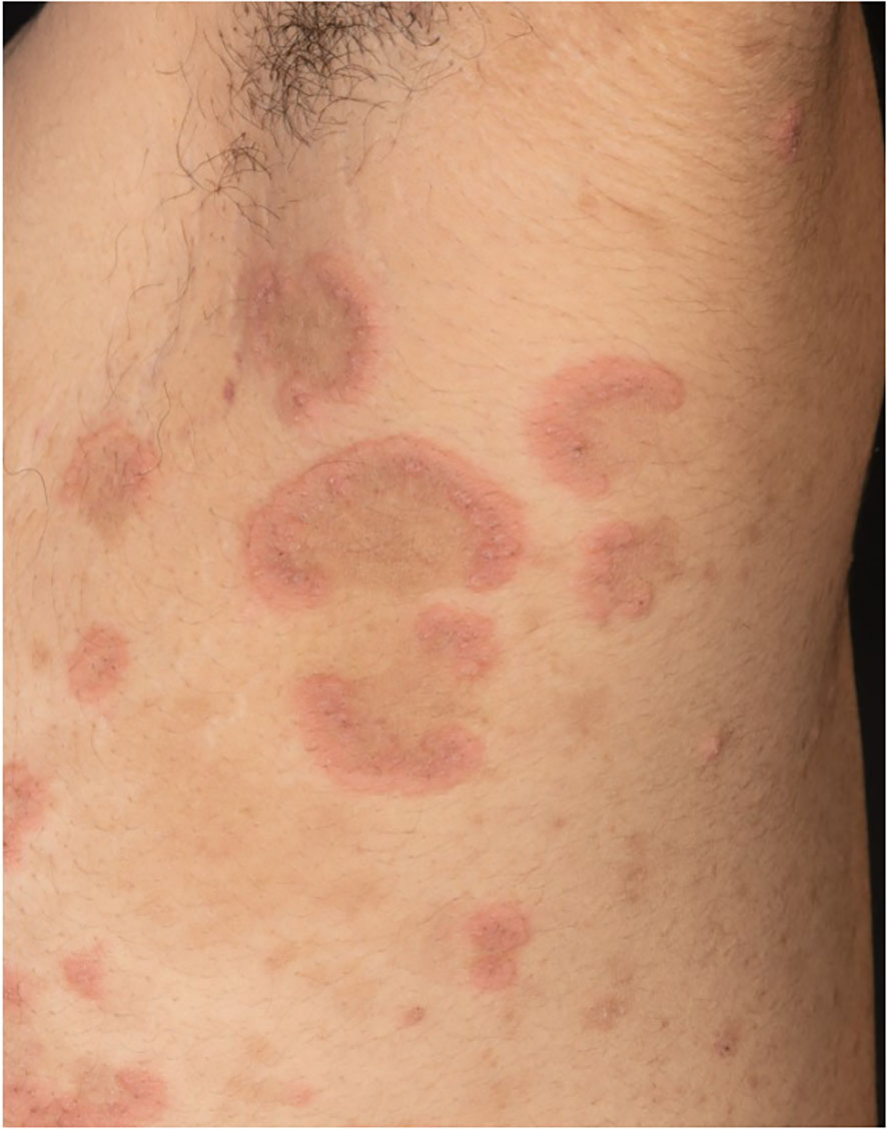
Multiple blisters, wheals, and erosions on the trunk and extremities.

The second case presents a 25-year-old male patient with sudden onset of multiple blisters, wheals, and erosions on his trunk and extremities (**Figure 2**). Based on increased BP180 antibody levels and linear C3 immunofluorescence along the basement membrane zone, BP was diagnosed. Treatment with mometasone furoate cream (topical) and prednisolone 0.5 mg/kg body weight (systemic) initially improved the skin lesions. Because of the severity of BP, we decided to use antibiotic as well as anti-inflammatory systemic therapy with dapsone and prednisolone. When the skin lesions relapsed upon tapering of prednisolone, systemic therapy with dapsone 100 mg/day p.o. was initiated. Both dapsone and mycophenolate mofetil were stopped after a few weeks by the patient himself, and consequently, his skin condition markedly deteriorated. He was hospitalized, and dapsone and mometasone furoate cream were restarted. A colonoscopy was performed due to incipient diarrhea, which incidentally detected a tumor in the left colon. The histological analysis revealed a concealed perforated adenocarcinoma of the colon and tumor staging cT4b N1b M1a with pulmonary metastases. An extended left hemicolectomy with partial liver resection, gastric fundus resection, omenectomy, left partial diaphragmatic resection, left partial lung lobe resection, and splenectomy were performed. A few days after tumor resection, the skin lesions healed, and the patient has since been in remission (for 6 months) without any medication.

**Figure 3:**
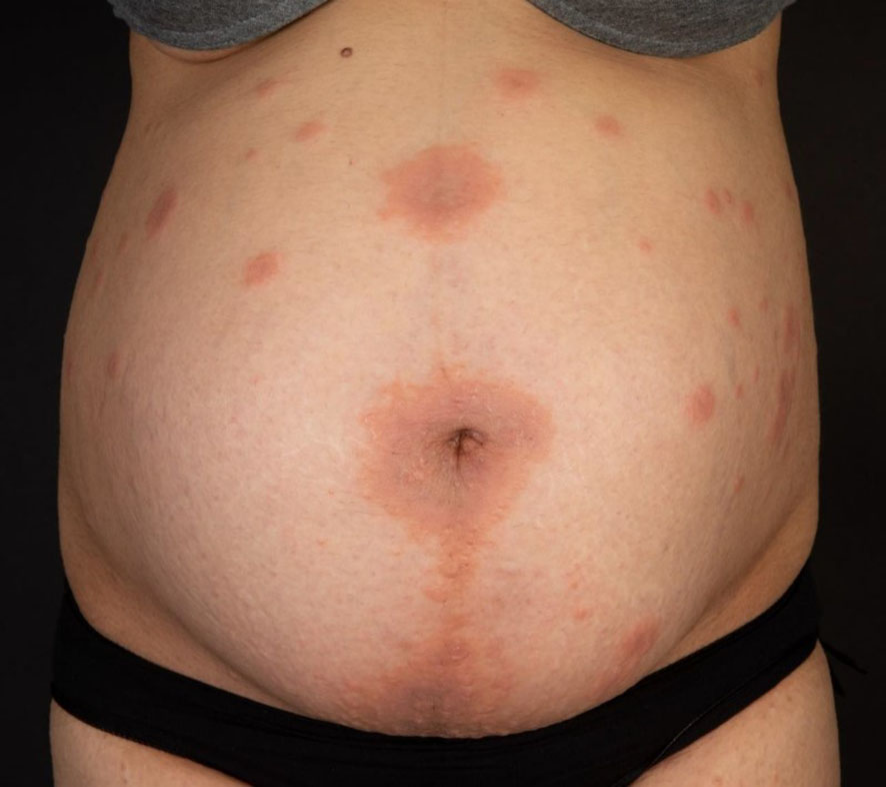
Widespread urticarial skin lesions on the trunk including the periumbilical region.

The third case shows a 34-year-old female patient in the 34th week of her third pregnancy, who had already developed skin manifestations diagnosed as polymorphic eruption of pregnancy during her second pregnancy (**Figure 3**). Two weeks prior to consultation, she had developed widespread urticarial skin lesions on her trunk including the periumbilical region. Based on the positive results of direct immunofluorescence, and histopathologic and immunoserologic examinations, she was diagnosed with pemphigoid gestationis. The patient received systemic treatment with prednisolone 0.5 mg/kg body weight and local therapy with methylprednisolone aceponate cream. Antipruritic therapy consisted of clemastine fumarate p.o. and polidocanol cream topically. Owing to pregnancy, the patient received prednisolone exclusively as a systemic therapy concomitant to local therapy. Following a moderate improvement of her skin lesions, the patient was discharged. Two weeks later, the patient continued to suffer from pruritus but without any new skin lesions. Subsequently, the patient did not present to our hospital again, so she and her baby were lost to follow-up.

**Figure 4:**
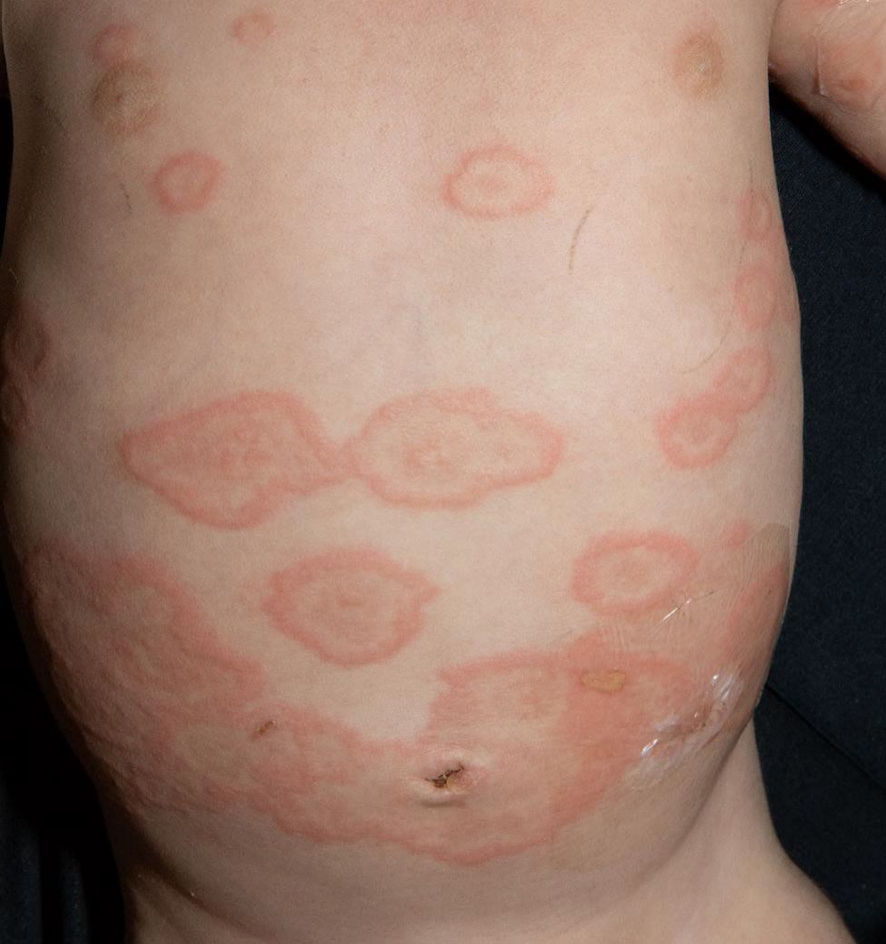
Multiple wheals and small erosions on the trunk and extremities.

The fourth and last case is a 3-month-old boy with sudden onset of multiple wheals and small erosions on his trunk and extremities (**Figure 4**). The therapy and course of disease are described in detail by Holtsche et al. (submitted for publication). Two weeks earlier, the patient has had conjunctivitis, followed by small wheals and plantar blisters. During further course, lesions aggravated and disseminated over the entire body surface. The direct immunofluorescence examination of a skin biopsy showed linear deposits of C3 at the basement membrane zone in addition to circulating anti-BP180 antibodies in blood serum. The mother reported an unremarkable pregnancy and showed neither skin lesions nor elevated BP180/230 antibodies. In summary, juvenile pemphigoid was diagnosed. Initially, the patient received local therapy with prednicarbate cream. As skin lesions progressed, systemic therapy was initiated with 2 mg/kg prednisolone p.o., which was gradually tapered over 3 months. Because of the age of the patient, we initially decided to use anti-inflammatory systemic therapy with prednisolone. In addition, the patient received a systemic therapy with IVIG (2 g/kg body weight) (Intratect^®^) and anti-CD20 antibody rituximab (12.5 mg/kg body weight). Complete disease remission was achieved after 4 months and therapy could be discontinued. Since then, the patient remained without evidence of disease and is without any other medication.

## Discussion

BP is classically seen in patients above the age of 70 and only rarely definite causes are found. Yet, exceptions need to be anticipated and pemphigoid disease is found in unusual circumstances. Although BP is not a common side effect of mRNA-COVID vaccination, there are some individual case reports describing this occurrence (4–12). To date, no cross-reactivity between SARS-CoV-2 antigens and BP180 could be demonstrated (13). However, both inception and exacerbation of BP have been described with other vaccinations as well and may be related to immunological disturbances during vaccine reaction, including the induction of autoimmune mechanisms.

The incidence of BP depends on ethnicity and is more common in Caucasians than in dark-skinned populations (14). On the other side, skin color may influence the course of BP disease: people of darker skin often show higher disease activity with higher BPDAI scores, higher levels of anti-BP180/230 IgG, and increased pruritus compared to a Caucasian population, as shown in a monocentric study (15). Moreover, people with darker skin types more commonly suffer from pigmentary changes even if control of disease activity has been achieved (16). The diagnosis of BP in dark skin may be challenging as the characteristic erythema is not easily discernible and diseases of colored skin are underrepresented in dermatologic textbooks (17).

Paraneoplastic BP has long been described, yet in recent meta-analyses, this connection has been debated. Approximately 11% of cases are suspected to be associated with malignancies. In this context, hemato-oncological diseases, renal cell carcinoma, or laryngopharyngeal cancer has been mainly described, but there are also rarer cases with other solid tumors (18). The pathogenesis of paraneoplastic BP has not yet been clarified in detail. However, it is suspected that antibodies against tumor-specific antigens cross-react with antigens of the basement membrane and, thus, cause blistering (19). As laminin-332 is present in many types of cancers, it is suggested that BP with antibodies against laminin-332 may be related to malignancies (20). On the other hand, no increased cancer risk has been observed in patients with BP (21). According to the current German S2k guideline, “diagnostics and therapy of bullous pemphigoid”, a tumor screening that excludes a paraneoplastic etiology is not routinely recommended (22). Nevertheless, a tumor screening should be performed if the patient does not show the usual clinical symptoms or if the disease course is refractory to therapy as described in our case.

Pemphigoid gestationis is a disease within the spectrum of BP with an incidence of 0.2/100,000 inhabitants/year. It occurs concomitantly with pregnancy, chorionic carcinoma, or bladder mole. The skin lesions usually disappear spontaneously after childbirth but may recur with a new pregnancy, perimenstrually, or with hormonal contraception. In addition to antipruritic treatment, therapy consists of high-potency topical glucocorticosteroids and may be escalated to systemic treatment with glucocorticosteroids (23). Skin lesions can also be observed in 10% of newborns due to placental transfer of maternal BP180 antibodies (24). Patients should refrain from hormonal contraceptives in the future to avoid disease recurrence. As BP180 is present in placental ectodermal chorionic and amniotic epithelium, pemphigoid gestationis may lead to placental insufficiency and preterm delivery or small-for-date babies (25). In our case, the patient already had pemphigoid gestationis during her second pregnancy, which was then misdiagnosed as polymorphous eruption in pregnancy.

Juvenile pemphigoid is a very rare disease with less than 100 described cases (26). Frequent acral involvement with self-limiting course and remission within 12 months is common (27). The relevant autoantibodies may be transferred from the mother via the placenta with clinically manifest or latent BP. These antibodies will disappear over the course of weeks to a few months after birth, necessitating only symptomatic, mainly topical treatment. Alternatively, autochthonous BP may be present with long-lasting disease and persistent autoantibodies requiring continued systemic immunosuppressive treatment.

The presented case series (**Table 1**) underline that BP should always be considered as an important differential diagnosis when it comes to blistering skin lesions regardless of the patient’s age and conditions.

**Table 1:** Overview table (IIF= indirect immunofluorescence, DIF= direct immunofluorescence).

	classification	immunological features	clinical presentation	therapy
**1. case**	**bullous pemphigoid**	BP180 antibodies BP230 antibodies IIF: linear IgG fluorescence along BMZ (monkey oesophagus) DIF: linear IgG & C3 immunofluorescence along BMZ	multiple erythematous, partly excoriated papules, blisters, erosions disseminated on the entire skin	1. prednisolone + dapsone 2. prednisolone + azathioprine 1. & 2. were discontinued due to side effects 3. IVIGs + mycophenolate mofetil, prednisolone topically: clobetasol proprionate cream
**2. case**	**paraneoplastic** **bullous pemphigoid**	BP180 antibodies linear C3 immunofluorescence along BMZ	multiple blisters, wheals and erosions on the trunk and extremities	1. prednisolone + dapsone (stopped by patient himself) 2. prednisolone + mycophenolate mofetil (stopped by patient himself) 3. dapsone 4. excision of adenocarcinoma of the colon topically: mometasone furoate cream
**3. case**	**pemphigoid gestationis**	BP180 antibodies linear C3 immunofluorescence along BMZ	widespread urticarial skin lesions on her trunk including the periumbilical region	Prednisolone topically: methylprednisolone aceponate cream
**4. case**	**juvenile** **bullous pemphigoid**	BP180 antibodies linear C3 immunofluorescence along BMZ	multiple wheals and small erosions on his trunk and extremities	1. prednisolone 2. IVIGs 3. rituximab topically: prednicarbate cream

Apart from the most common type of BP, other manifestations and disease subsets are encountered. These cases highlight the various rare manifestations of pemphigoid disease that first need to be recognized and treated accordingly.

## Data availability statement

The raw data supporting the conclusions of this article will be made available by the authors, without undue reservation.

## Ethics statement

Written informed consent was obtained from the individual(s), and minor(s)’ legal guardian/next of kin, for the publication of any potentially identifiable images or data included in this article.

## Author contributions

LR wrote the manuscript. LS, YF, and CB took part in drafting, revising, and critically reviewing the article. MS was responsible for the conceptualization as well as project administration and reviewed the manuscript. All authors contributed to the article and approved the submitted version.
